# Advances in Carbon Nanotubes: Synthesis, Properties, and Cutting-Edge Applications

**DOI:** 10.3390/nano15201595

**Published:** 2025-10-20

**Authors:** Guohai Chen, Dai-Ming Tang

**Affiliations:** 1 Nanocarbon Material Research Institute, National Institute of Advanced Industrial Science and Technology (AIST), Tsukuba Central 5, 1-1-1 Higashi, Tsukuba 305-8565, Japan; 2 Research Center for Materials Nanoarchitectonics (MANA), National Institute for Materials Science (NIMS), Tsukuba 305-0044, Japan; 3 Institute of Pure and Applied Sciences, University of Tsukuba, Tsukuba 305-8571, Japan

Carbon nanotubes (CNTs) have remained at the forefront of nanoscience for more than three decades, owing to their unique cylindrical structures, exceptional physical, chemical, and mechanical properties, and broad potential in electronics, energy, composites, and biomedical applications. Significant advances have been achieved in scalable synthesis, structural control, and multifunctional integration. More recently, the rapid development of artificial intelligence (AI) and machine learning (ML) methodologies has opened new opportunities to optimize CNT growth, tailor structures, and predict performance with unprecedented precision.

This Special Issue of *Nanomaterials*, “Advances in Carbon Nanotubes: Synthesis, Properties, and Cutting-Edge Applications,” assembles ten contributions from several research groups worldwide [1,2,3,4,5,6,7,8,9,10]. The collection comprises five research articles and five review articles that span fundamental synthesis studies, advanced characterizations, and diverse application demonstrations, reflecting both the maturity of the field and the emergence of new directions.

## 1. Synthesis and Structural Engineering

A central topic in CNT research remains the precise control of synthesis and its structures. Several contributions focus on refining CNT growth strategies and tailoring their structures for targeted applications. Shimizu et al. [1] introduced a chromatographic approach to effectively assess CNT–organic interactions, enabling improved CNT dispersibility in suspensions. Their study provides a versatile platform for understanding interfacial chemistry, which is critical for tailoring CNT compatibility with polymers, solvents, and functional molecules in advanced applications. Matsumoto et al. [2] reported the direct growth of CNTs on aluminum nitride substrates, demonstrating a marked improvement in composite thermal conductivity with reduced filler ratios. This approach represents a significant advancement in the design of thermally conductive materials, with potential implications for a wide range of applications. Nwanno et al. [3] fabricated copper-filled vertically aligned CNTs (VACNTs) directly on copper foil substrates, achieving enhanced field emission performance. Their findings demonstrate the viability of thin copper substrates in creating dense and highly conductive copper-filled VACNT arrays for advanced electronic and nanoelectronics applications.

## 2. Properties from Nano to Macro

Translating the intrinsic properties of CNTs into macroscopic performance remains another core challenge. Xiang et al. [4] comprehensively review the electrical properties and measurement techniques of CNTs, spanning from individual nanotubes to macroscopic assemblies. They highlight the difficulties in transferring the electrical properties from nanoscale to bulk materials and proposed strategies for future research and development directions to boost the electrical conductivity of CNT assemblies. Rezaee et al. [5] fabricated single-walled CNT (SWCNT) thin films using the floating catalyst chemical vapor deposition method and systematically explored their electrical characteristics after acid treatment through Hall effect measurements. Their study offers new insights into the correlation between structural, electrical, and optical properties, contributing to a deeper understanding of structure–property relationships.

## 3. Application-Oriented Developments

The versatility of CNTs is further illustrated through application-focused contributions. Ishihara et al. [6] developed a purge-free, actuator-driven formaldehyde gas sensor based on SWCNT chemiresistors. By leveraging periodic actuation, the sensor achieved reliable detection of formaldehyde gas over a wide concentration range (0.05–15 ppm) with excellent selectivity over other volatile organic compounds and stability, underscoring the utility of CNTs in environmental monitoring. Almansoori et al. [7] present a comprehensive review of CNT/graphene-reinforced ceramics, detailing how these hybrid materials can achieve superior toughness, hardness, and thermal stability compared to conventional ceramics, while also highlighting challenges in processing, scalability, and interfacial compatibility that must be addressed for practical applications. Alfei et al. [8] review antimicrobial CNT composites, emphasizing their potential in medical, packaging, and environmental applications. They explore how CNTs can inhibit microbial growth while also addressing concerns of cytotoxicity, environmental impact, and regulatory acceptance, thereby offering balanced insights into performance and safety considerations. Snowdon et al. [9] review strategies to enhance product durability of CNT-based materials, focusing on synergistic molecular assembly, intrinsic and engineered self-repair, and advanced characterization techniques. By identifying current challenges and future research frontiers, the review underscores that the creation of truly durable materials depends on an integrated understanding of how to build, repair, and precisely measure CNT-based systems.

## 4. Emerging Trends and AI Integration

A particularly exciting frontier is the integration of AI and ML into CNT research. Chen et al. [10] review the role of ML as a “catalyst” for accelerating CNT research, covering areas such as synthesis optimization, characterization, property prediction, and application development. The review highlights how data-driven approaches can close the loop between experiment and theory, potentially enabling autonomous laboratories for discovery and design of CNT-related research. Such approaches are likely to become increasingly important for accelerating innovation in nanomaterials science.

## 5. Outlook

Taken together, the contributions in this Special Issue capture the dynamic progress of CNT research. They reveal how advances in synthesis and structural engineering, the translation of properties from nano to macro scales, and the expansion of applications into sensing, composites, and biomedicine are converging to unlock the full potential of CNTs. At the same time, emerging AI-driven methodologies are reshaping how the field approaches discovery and optimization.

We hope this collection not only highlights the representative advances in CNT research but also inspires future interdisciplinary innovation. In particular, the integration of AI with CNT science has the potential to redefine material design, accelerate translation into real-world technologies, and drive the next breakthroughs in nanomaterials.

Looking forward, key priorities include bridging the gap between nanoscale performance and scalable industrial implementation, developing sustainable and cost-effective synthesis routes, integrating AI-guided design into mainstream workflows, and addressing safety, environmental, and regulatory considerations to support broader commercialization. With these combined efforts, CNTs will continue to inspire and enable transformative technologies in the years ahead.

## Data Availability

Not applicable.
